# YOU’RE THE ONLY PERSON THERE FOR THEM: LEVERAGING HOME HEALTH AIDES’ EXPERTISE TO SUPPORT TOTAL CLIENT HEALTH

**DOI:** 10.1093/geroni/igz038.791

**Published:** 2019-11-08

**Authors:** Emily C Franzosa

**Affiliations:** CUNY Graduate School of Public Health and Health Policy, New York, New York, United States

## Abstract

Formally, home health aides provide physical, non-clinical care to support the health and safety of older and disabled individuals. But in practice, both workers and clients report that the often unrecognized relational care aides provide is also central to clients’ well-being. In focus groups with New York City-based home health aides, aides described their conception and delivery of “total” care, which included specific and deliberate cognitive, emotional and social strategies to support clients’ mental and physical well-being. However, since this work was not included in formal care plans and invisible to those outside the care team, aides felt unprepared and unsupported in performing it. Realigning the definition of home care to address “total” care and better integrating aides into the care team has the potential to both improve job satisfaction and patient care.

